# Expression and purification of the modification-dependent restriction enzyme BisI and its homologous enzymes

**DOI:** 10.1038/srep28579

**Published:** 2016-06-29

**Authors:** Shuang-yong Xu, Pernelle Klein, Sergey Kh. Degtyarev, Richard J. Roberts

**Affiliations:** 1 New England Biolabs, Inc. 240 County Road, Ipswich, MA 01938, USA; 2 SibEnzyme Ltd., 2/12 Ak, Timakov Street, Novosibirsk 630117, Russia

## Abstract

The methylation-dependent restriction endonuclease (REase) BisI (G^m5^C ↓ NGC) is found in *Bacillus subtilis* T30. We expressed and purified the BisI endonuclease and 34 BisI homologs identified in bacterial genomes. 23 of these BisI homologs are active based on digestion of ^m5^C-modified substrates. Two major specificities were found among these BisI family enzymes: Group I enzymes cut GCNGC containing two to four ^m5^C in the two strands, or hemi-methylated sites containing two ^m5^C in one strand; Group II enzymes only cut GCNGC sites containing three to four ^m5^C, while one enzyme requires all four cytosines to be modified for cleavage. Another homolog, Esp638I cleaves GCS ↓ SGC (relaxed specificity RCN ↓ NGY, containing at least four ^m5^C). Two BisI homologs show degenerate specificity cleaving unmodified DNA. Many homologs are small proteins ranging from 150 to 190 amino acid (aa) residues, but some homologs associated with mobile genetic elements are larger and contain an extra C-terminal domain. More than 156 BisI homologs are found in >60 bacterial genera, indicating that these enzymes are widespread in bacteria. They may play an important biological function in restricting pre-modified phage DNA.

Type IIM and Type IV restriction endonucleases (REases) cleave only modified DNA and are inactive on unmodified DNA^1^. They have evolved in the arms race between bacteria and bacteriophages by restricting phage with modified bases in their genomes (reviewed in ref. 2). Type IIM REases such as DpnI (G^m6^ATC)^3^, BisI (G^m5^CNGC)^4^, GlaI (R^m5^CGY)^5^, and MspJI (^m5^CNNR 9/13)^6^ cleave modified sites within or close to their recognition sequences at defined positions^7^. In contrast, Type IV REases cleave modified sites randomly and often at a great distance from their recognition sequences (e.g. EcoK_McrBC (R^m5^C N_(40–3000)_ R^m5^C)^8^, SauUSI (S^m5^CNGS)^9^, ScoA3McrA (phosphorothioated sites)^7^,^10^. Type IIM and IV REases are useful tools for analyzing ^m5^C-modified sites in mammalian DNAs since hyper-methylation of CpG sites can alter gene expression (e.g. in ref. 11). GlaI has been used to digest hypermethylated cancer genomic DNA (gDNA) and following ligation of adaptors to the digested fragments, the cancer marker region can be selectively amplified and sequenced^11^. GlaI has also been used in a real time activity assay for the human DNA methyltransferase (MTase) DNMT1^12^. The methylation-dependent REases (MDRE) McrBC and FspEI (C^m5^C) can be used in qPCR or digital PCR applications to monitor changes in epigenetic markers of clinical DNA samples^13^. DpnI is used to destroy the wild-type (WT) template after PCR, thus reducing the background in PCR-directed mutagenesis experiments (G^m6^ATC sites in the template methylated by the *E. coli* Dam methylase). MspJI-seq (NGS sequencing of an MspJI-cleaved library) has been used to map modified sites in the *Arabidopsis* genome^14^. While most methylation-dependent REases can cut both ^m5^C- and ^hm5^C-modified DNA, REases such as PvuRts1I that prefer to cleave ^hm5^C-modified DNA are also found in Nature^15^,^16^. Eco94GmrSD, however, prefers to cleave ^hm5^C-modified and glucosylated ^hm5^C T4 DNA; GmrSD digests ^m5^C-containing DNA and unmodified DNA poorly^17^,^18^.

BisI was first discovered and purified from a bacterial source *Bacillus subtilis* T30 and it cleaves GCNGC sites when two to four modified ^m5^C residues are present in its recognition sequence^4^,^19^. The BisI homologues PkrI and GluI, however, require three to four modified ^m5^C in GCNGC for enzymatic activity^20^,^21^. The enzyme yield and purity of all three enzymes are relatively low from the native bacterial sources making their cost prohibitive for widespread applications in diagnostic qPCR and NGS applications. Highly purified enzymes are also a prerequisite for further enzyme characterization and for structure analysis.

The goal of this work was to provide more modification-dependent REases for molecular biology and diagnostic applications. Here we report the cloning and expression of the BisI restriction enzyme gene in *E. coli*. BisI is the prototype for a new family of methylation-dependent REases since BisI does not share any significant amino acid (aa) sequence homology to the other known Type IIM restriction enzymes such as the DpnI, MspJI, McrBC, Mrr, McrA, ScoA3McrA or SauUSI families. By using the BlastP server at NCBI to search genome sequences in GenBank, we identified over 150 BisI homologs in bacterial genomes with 17% to 100% aa sequence identity. We cloned/expressed some of these genes in *E. coli* and identified 23 active BisI homologs with varying degrees of ^m5^C requirement in cleaving modified GCNGC or its variant sites. We found one BisI homolog (Esp638I) with a unique specificity G^m5^CS ↓ SG^m5^C, but also capable of relaxing its specificity to RCN ↓ NGY. We also determined that some BisI family enzymes cleave hemi-methylated sites with two ^m5^C in one DNA strand. In addition, we found two BisI homologs with degenerate specificities cleaving unmodified DNA.

## Results

### Searching for the BisI restriction gene (*bisIR*) in the *B. subtilis* T30 genome

The *B. subtilis* T30 genome was sequenced using a Pacific BioSciences RSII sequencer and the sequences were assembled into a single circular contig of 4.03 Mbp with 4,138 predicted genes (GenBank accession number CP011051)^19^. Initially, one small putative HNH endonuclease gene (Bis30_20225, 124-aa, a.k.a. BspT30 HNH endonuclease) and one phospholipase D (PLD) family endonuclease gene (Bis30_09935, 221-aa) were considered as candidate genes for BisI. The partially purified gpBis30_20225 displayed strand-specific and sequence-specific DNA nicking activity with the specificity 5′ RCG ↓ GT 3′ in Mg^2+^ buffer and a more relaxed specificity under star conditions^22^. The gpBis30_20225 nicking specificity is similar to that of phage Gamma HNH endonuclease (N.ϕGamma, 5′ CG ↓ GT 3′) and gp74 of phage HK97 (5′ GCG ↓ GT 3′) that are thought to be involved in nicking of the *cosN* site and in DNA packaging^22^,^23^,^24^. We next evaluated a second putative endonuclease encoded by Bis30_09935, a predicted PLD family endonuclease. The gene was expressed under the control of an inducible T7 promoter (pET21a), but no specific endonuclease activity was detected on either modified or unmodified DNA (data not shown).

Further sequence analysis of the *B. subtilis* T30 genome revealed one small ORF (Bis30_20260, adjacent to an inactive C5 MTase (Bis30_20265), which proved to be the gene for BisI. IPTG-induced *E. coli* cell extracts expressing both ORFs or the single ORF, Bis30_20260, in pET21a under the T7 promoter, displayed site-specific and modification-dependent endonuclease activity on the modified substrate pBRFM (G^m5^CNGC or G^m5^CNG^m5^C) (data not shown). Inspection of the *bisIR* gene indicates that there are two possible start codons in the gene: the longer ORF (178 aa) with an ATG start codon (with a poor ribosome binding site upstream) and a shorter ORF with a TTG start codon encoding a 168-aa protein with a Shine-Dalgarno sequence. To assess which codon was the likely start for the active BisI endonuclease we cloned both versions of the gene (PCR products) into the IMPACT expression vector pTYB1 as fusions with an intein and a chitin-binding domain (CBD). The long form (178 aa) produced only small amounts of active protein, whereas the short form generated a moderate amount of protein after DTT/intein cleavage from chitin columns. It was concluded that BisI restriction gene is most likely 504 bp encoding a 168-aa BisI endonuclease with a predicted molecular mass of 19.4 kDa. The recombinant BisI purified from *E. coli* appeared to be ~21 kDa with five additional aa residues (LEGSS, vector-derived) at the C-terminus after DTT/intein cleavage. The BisI enzyme was further purified from a Heparin column (data not shown). Run-off sequencing of the cleavage products of BisI-digested pBRFM indicated that the cleavage site is the same as reported for the native enzyme G^m5^C ↓ NGC ( ↓  indicating the cleavage position of the strand shown, data not shown)^4^. BisI does not show any aa sequence similarity to known Type IIM or Type IV REases and therefore forms its own family. Multiple attempts to sequence the partially purified native BisI enzyme failed to obtain the N-terminal aa sequence (Too P, Dalton M, Benner J, SYX, unpublished results). Therefore, the bona fide start codon in the native *B. subtilis* T30 strain is still unknown. *Bacillus subtilis* subsp. Spizizenii strain W23 (ATCC 6633) encodes a protein (Bsu6633_05694) identical to BisI, but its activity has not been tested. Other highly similar BisI homologs ranging from 67% to 88% aa sequence identity are also found in sequenced *Bacillus subtilis* (WP_024573623) and *Bacillus* sp. genomes (WP_053358749, WP_035432006) that remained to be evaluated.

### Cloning/expression and purification of BisI homologs

We used the BisI aa sequence to query GenBank using BlastP and found over 156 homologs (103 homologs with >25% aa sequence identity; E value <0.004; 53 homologs with 19% to 25% aa sequence identity, E value >0.005) in March 2016 in more than 60 bacterial genera and mega-genome sequences (data not shown). Thus, BisI homologs are present in many bacteria.

One homolog is the Eco15I endonuclease (active, see below), which has an N-terminal 160-aa with significant similarity to BisI. However, it also contains an extra C-terminal domain of ~130-aa that shows significant similarity to some putative HNH endonucleases (COG1403 and COG 3183, Pfam01844). In some bacteria, the homologs of the C-terminal domain of Eco15I exist as separate small proteins 88 to 129-aa long whose function remains to be determined. A total of 34 Eco15I homologs (263 to 294-aa residues long) were found in GenBank mainly among Gram-negative bacteria, including some pathogens. In the shot-gun sequence of the DNA fragment encoding Eco15I, the restriction gene is located next to an XRE family transcription regulator, two putative DNA transposases (COG1662 and COG3677) and an IS1 protein InsA.

**Figure 1:**
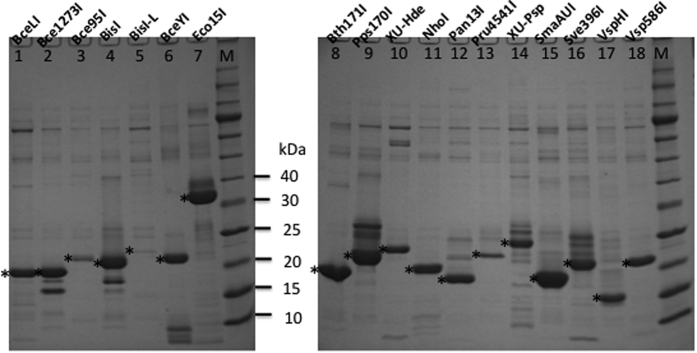
SDS-PAGE analysis of partially purified BisI family enzymes. Expected enzyme products are marked by “*” (lanes 1–18) from chitin columns following DTT/intein cleavage. M, protein size ladder. See Tables 1, 2, 3 for description of the enzymes.

We evaluated some of the homologs for modification-dependent restriction activity. These BisI homologous genes were cloned into pTYB1 as fusions containing an intein and a CBD so that the target protein could be purified quickly from affinity chitin columns and DTT/intein cleavage (one BisI homologs, Rfl17I, was 6xHis-tagged at the N-terminus and was purified from Ni-NTA affinity column, data not shown). Figure 1 shows some partially purified BisI homolog proteins. The purified enzymes were used to digest pBRFM which contains two and three modified ^m5^C in GCNGC sites or phage XP12 DNA in which all C residues are replaced by ^m5^C. We also used DNA duplex oligos with two, three, or four ^m5^C in a GCWGC site for enzyme digestion (see below). BisI-L (long form, 178 aa) was poorly expressed and showed a low activity (Fig. 1, lane 5). The smallest active enzyme is VspHI (Fig. 1, lane 17) with 150 aa, and the largest homolog is Eco15I (Fig. 1, lane 7) with 290 aa. Tables 1, 2, 3, [Table t1], [Table t2], [Table t2], [Table t2], [Table t2], [Table t3] list all the active BisI homolog enzymes.

### Bce95I recognition sequence and cut site determination

**Figure 2:**
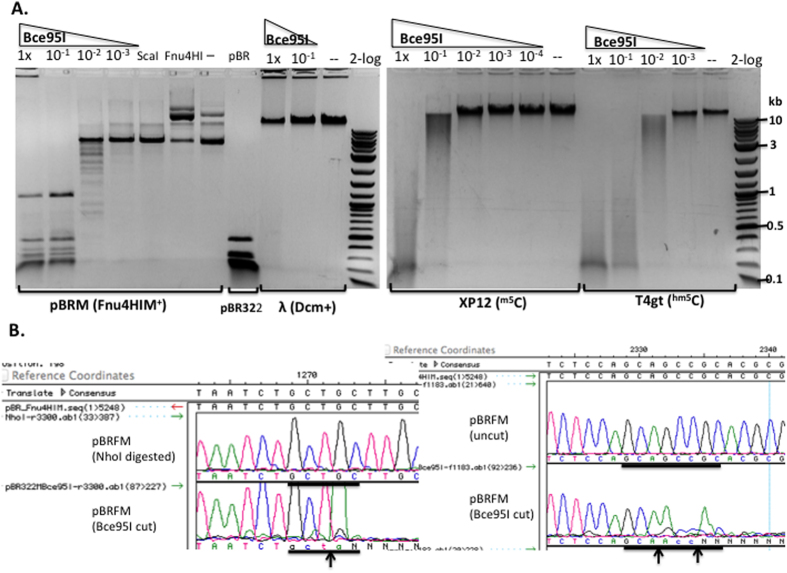
Bce95I activity assay and run-off sequencing to determine cut sites. (**A**) Bce95I digestion of pBRFM, phage λ, XP12, and T4gt DNAs. Bce95I enzyme dilution factors are indicated on the top of each lane. ScaI, ScaI-linearized pBRFM; Fnu4HI, Fnu4HI-digested pBRFM (note: the plasmid is resistant to digestion due to methylation); pBR, Fnu4HI-digested pBR322; “--”, uncut DNA; 2-log, 2-log DNA ladder. (**B**) DNA run-off sequencing of the Bce95I cleavage site G^m5^CTGC and G^m5^CAG ^m5^CCGC of pBRFM. The up arrow indicates the bottom strand (template) is cleaved. The extra A peak indicates a cut in the bottom strand template (indicated by ↑ arrow). The extra T peak indicates a cut in the top strand (indicated by ↓ arrow). The color-coded sequence traces are: A (green), T (red), C (blue), G (black). The extra A trace (or T on the opposite strand) was added at the end of the cleaved template by the Taq DNA polymerase (template-independent terminal nucleotide transferase activity).

We chose Bce95I (a BisI-like isoschizomer) to digest the three modified DNA substrates (pBRFM, phage XP12, and T4gt). Bce95I completely digested ScaI-linearized pBRFM DNA (Fig. 2A), with a specific activity estimated at 5 × 10^3 ^units/mg protein. Bce95I partially digested phage XP12 and T4gt DNAs, but did not show activity on λ DNA (C^m5^CWGG, M.Dcm^+^). Similar results were obtained for another BisI-like isoschizomer BceYI (data not shown). The reason for the inhibition of Bce95I and BceYI restriction activity on the heavily modified phage DNAs is unknown. However, one possibility is that the enzymes may bind very tightly to the modified sites, resulting in slow enzyme turn over, or the enzyme may remain bound to the cleavage products, thereby preventing enzyme turn over. Run-off sequencing of Bce95I-digested pBRFM DNA showed that the recognition sequence and cut site are the same as BisI; sites with two ^m5^C (G^m5^CT ↑ GC, Fig. 2B, left) and three ^m5^C (G^m5^CA ↑ G^m5^CC ↑ GC, Fig. 2B, right) are cleaved by Bce95I. A duplex DNA oligo containing four ^m5^C (GCWGC) is also cleaved by Bce95I (see below).

### NhoI recognition sequence and cut site determination

**Figure 3:**
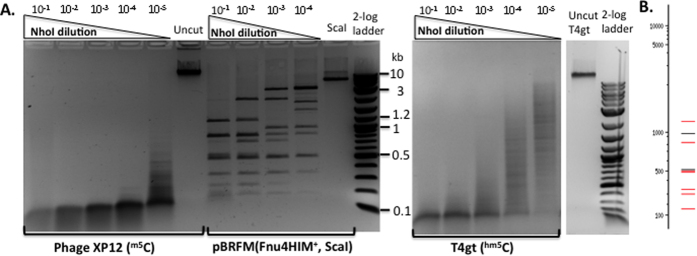
NhoI endonuclease activity assays. (**A**) NhoI digestion of phage XP12 (all ^m5^C), pBRFM (ScaI-linearized, three ^m5^C in G^m5^CNG^m5^CNGC), and T4gt DNA (^hm5^C). Enzyme dilution factors are indicated on the top of each lane. (**B**) A theoretical digest of pBRFM (NEBcutter)^25^ by ScaI and another enzyme cleaving GCNGCNGC (expected sizes in bp: 1168, 966, 828, 515/504/484, 333/297, 153).

**Figure 4:**
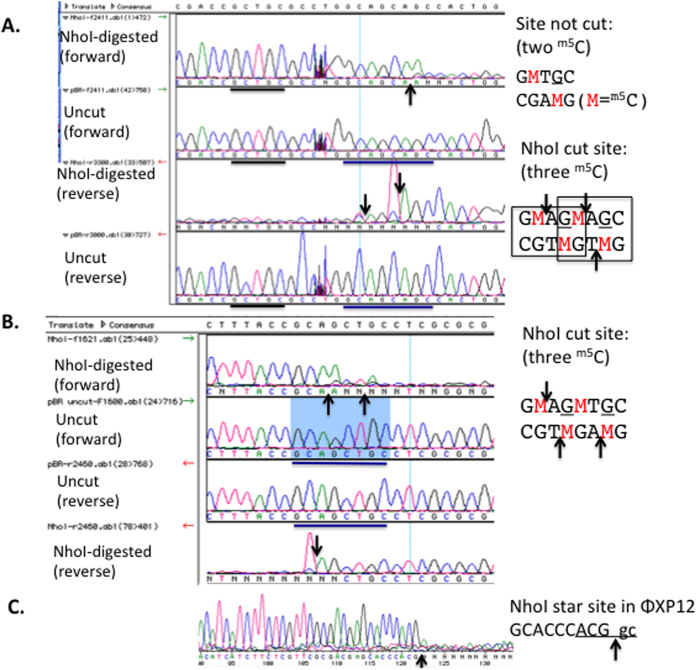
Run-off sequencing to determine NhoI cut sites. DNA run-off sequencing at G^m5^CTGC (incubated with NhoI endonuclease, but this site not digested) and G^m5^CAG^m5^CAGC sites of NhoI-digested plasmid pBRFM (M.Fnu4HI). The G^m5^CTGC site indicated by the black bar contains two ^m5^C. The G^m5^CAG^m5^CAGC site indicated by the blue bar contains three ^m5^C.

We next chose NhoI for further characterization. Figure 3A shows that NhoI cleaved phage XP12 DNA with the highest activity (~2 × 10^5 ^units/mg protein), but the activity is 50 to 100-fold lower on T4gt DNA which contains ^hm5^C-modified bases. To determine the cut site, NhoI-digested pBRFM was used as a template for DNA run-off sequencing. Figure 4A,B show that G^m5^CNG^m5^C sites with three ^m5^C are cleaved, but G^m5^CNGC sites with two ^m5^C on two strands are not digested. On plasmid substrates with three modified ^m5^C residues, G^m5^CWG^m5^C sites appeared to be cleaved more completely than a G^m5^CSG^m5^C sites (data not shown). NhoI only cuts those GCNGC sites on pBRFM where three ^m5^C are present in G^m5^CNG^m5^CNGC sequences. Those sites were nearly completely digested at 10-fold enzyme dilution compared to a theoretical digestion by NEBCutter (Fig. 3B)^25^. Direct sequencing of XP12 DNA digested by NhoI (NEB buffer 2.1 and high enzyme concentration) also identified a star site A^m5^CGG^m5^C (Fig. 4C), consistent with the small digested fragments around 100 bp that constitute the final products. The relaxed specificity of NhoI can be summarized as R^m5^CNG^m5^C (R = A or G, three to four ^m5^C). Relaxed sequence recognition by NhoI can also occur in other positions under star conditions (RJR, unpublished result).

### Digestion of DNA duplex oligos with four ^m5^C (symmetric methylation), three ^m5^C (asymmetric methylation), and two ^m5^C (hemi-methylated) by BisI, NhoI, and other enzymes

**Figure 5:**
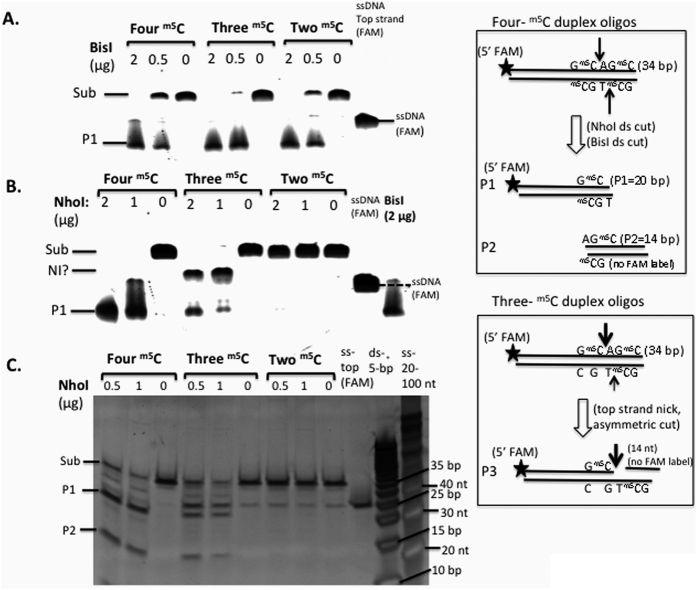
Digestion of ^m5^C-modified duplex oligos (GCWGC site) by BisI or NhoI. The 5′-FAM-labeled top strand contains two ^m5^C bases (G^m5^CAG^m5^C) and the bottom strands contain either two (G^m5^CTG^m5^C), one (G^m5^CTGC, internal C methylated), or no ^m5^C bases (GCTGC), respectively. Thus, the annealed oligos contain a total of four, three, or two ^m5^C. P1 (20 bp) and P2 (14 bp) are the cleavage products. P3 is a possible top-strand nicked intermediate (NI) due to asymmetric nicking of the top strand. The substrate (sub, 34 bp), P1, and P3 were detected by FAM fluorescence imaging. (**A**,**B**). BisI- and NhoI-digested duplex oligos (four, three, or two ^m5^C) analyzed on a 15% PAG-urea denaturing gel. **C**. Partial digestion of the duplex oligos by NhoI and the DNA products were analyzed on a 20% TBE (non-denaturing) gel, stained by SYBR Gold and imaged by fluorescence imaging. The 5-bp dsDNA size marker (Fermentas) and the single-stranded oligos (IDT, 20–100 nt) were used to estimate the size of the cleavage products.

In the next set of experiments, we asked whether hemi-methylated sites could serve as substrates for BisI and NhoI. We used a set of ^m5^C-modified oligos (GCWGC) as the substrates (see Materials and Method for the oligo sequences). Figure 5A shows the results of BisI digestion of duplex oligos with four, three or two ^m5^C (hemi-methylated). BisI was able to digest all three substrates including the hemi-methylated oligos. NhoI cleaved modified oligos containing four ^m5^C better than oligos with three ^m5^C residues, in agreement with the digestion results of modified plasmid and phage XP12 DNA (all ^m5^C). But NhoI generated some cleavage intermediate, possibly top-strand nicked product (see the diagram in a box in Fig. 5). NhoI failed to digest the hemi-methylated duplex oligos.

**Figure 6:**
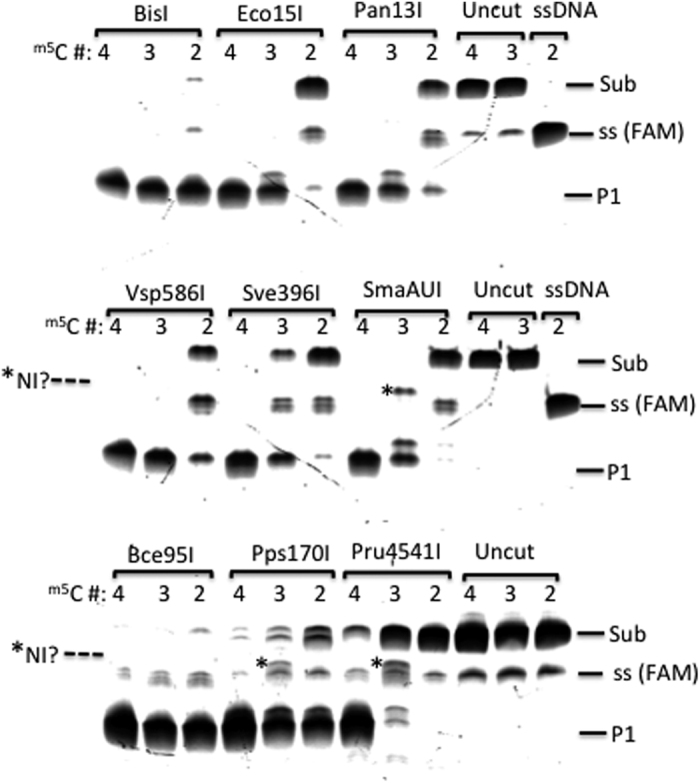
Digestion of DNA duplex oligos with four, three, or two ^m5^C in GCWGC by the indicated REases. Sub, modified duplex DNA substrate; P1, FAM-containing cleavage product (20 bp); NI?, possible top-strand nicking intermediate; ss, single-stranded (top strand) FAM-labeled DNA (34 nt).

We also tested a number of other BisI homolog enzymes for their ability to cleave the modified oligos. The results are shown in Fig. 6. The duplex oligos with three or four ^m5^C can serve as good substrates for BisI, Bce95I, Vsp586I, Eco15I, Pan13I, Pps170I, SmaAUI, and Sve396I. Pru4541I shows a strong substrate preference for oligos with four ^m5^C. BisI and Bce95I cleaved the hemi-methylated oligos efficiently. Vsp586I and Pps170I showed moderate endonuclease activity on the hemi-methylated substrate. Eco15I, Pan13I, SmaAUI, Sve396I, and Pru4541I showed poor activity on the hemi-methylated duplex DNA. Similar to NhoI, Pru4541I also accumulated possible nicking intermediate (top-strand) on oligos with three ^m5^C (asymmetric methylation). Interestingly, Vsp586I also accumulated a possible nicking intermediate (top strand) on the hemi-methylated DNA. The partial nicking activity of BisI family enzymes on asymmetrically modified DNA needs to be further investigated.

In summary, the active BisI family enzymes (BisI-like) that can cut two, three, or four ^m5^C in GCNGC sites are shown in Table 1. Those enzymes (NhoI-like) that require three or four ^m5^C for efficient cleavage are shown in Table 2. Bce1273I and Bth171I were also able to cleave pBR322 (M.Dcm^+^) and have relaxed specificities (see below, Table 3). Some homologs appeared to be inactive although proteins were made (alternatively, the inactivity may be due to the lack of appropriately modified sites in the substrates tested). Those inactive ones are listed in Supplementary Table S1.

### Screening for BisI family enzymes that exclusively cut GCNGC sites with four ^m5^C

In the first batch of screening, we found 14 active BisI homologs able to cleave pBRFM (two to three ^m5^C) or pBR322 (M.Dcm^+^) (Tables 1, 2, 3, [Table t1], [Table t2], [Table t2], [Table t2], [Table t2], [Table t3]). In a second screening we focused on homologs with a lower similarity (17% to 28% aa sequence identity) to BisI and the results are shown in Tables 1, 2, 3, [Table t1], [Table t2], [Table t2], [Table t2], [Table t2], [Table t3] and Supplementary Table 1. We expressed and purified 14 more homologs (Supplementary Figure S1A). Two had poor protein yields and no detectable activity, one had low yield and no detectable activity, and 8 homologs were active in cleaving pUCM, pBRFM, and phage XP12 DNA. Most importantly, three enzymes showed strong activity on phage XP12 DNA, but poor activity (or only nicking activity) on pUCM (Supplementary Figure S1, panels B and C). To confirm the activity of these three enzymes, modified duplex oligos were assayed by restriction digestion. Supplementary Figure S2, panel A shows that MbaR4I and SqiI are active in cleaving duplex oligos GCWGC with four ^m5^C (left). MbaR4I is also active in cleaving the substrate with three ^m5^C at reduced efficiency (middle); while SqiI shows poor activity on this substrate (right). To confirm the above results, we also digested the duplex oligos GCWGC (4, 3, or 2 ^m5^C) with MbaR4I or SqiI and detected the cleavage product (P1, P2) by SYBR Gold staining. MbaR4I was active on duplex oligos GCWGC with three to four ^m5^C and SqiI was active on the four ^m5^C substrate only. As expected, MbaR4I and SqiI were inactive in cleaving hemi-methylated substrate with two ^m5^C on the top strand. Five other BisI homologs were capable of cleaving hemi-methylated duplex oligos G^m5^CAG^m5^C (Supplement Figure S2C). Sde240I cleaved GCNGC with three to four ^m5^C sites better than two ^m5^C, but it also has a low activity on hemi-methylated duplex oligos. It was concluded that SqiI endonuclease prefers to cut G^m5^CWG^m5^C sites with four ^m5^C residues as indicated.

### Multiple amino acid sequence alignment for BisI family enzymes

Multiple aa sequence alignment of BisI/BceYI/Bce95I-like enzymes (cleavage of two to four ^m5^C sites) and NhoI/Pru454I/SqiI-like enzymes (cleavage of three to four ^m5^C sites) are shown in Supplementary Figures S3 and S4. The predicted secondary structures contain the typical restriction enzyme fold (αβββαβ) that harbors the catalytic residues D or E, D-X_(10–12)_-QxK for metal ion (Mg^2+^) binding and catalysis. Five conserved aa residues (D, E, and K, candidates for catalytic residues) are shown above the aa sequence alignment for the BisI/BceYI/Bce95I-like enzymes. The Mrr-like catalytic site found in the NhoI/Pru4541I/SqiI-like enzymes is a variant of the conserved PD-D/ExK catalytic site (the catalytic site PD-X_10–21_-D/ExK or PD-X_10–21_-D/ExE that is shared by >70% of all REases)^26^,^27^,^28^,^29^. The importance of these predicted catalytic residues remain to be investigated.

### Analysis of the Esp638I recognition sequence and cut site

The unexpected results of Esp638I digestion (active on phage XP12 DNA, poor activity on M.Fnu4HIM-modified plasmid or methylated duplex oligos G^m5^CWG^m5^C) prompted us to investigate the Esp638I specificity further. PBR322 was methylated by M.SssI (CG converted to ^m5^CG), M.CviPI (GC converted to G^m5^C), M.HhaI (GCGC converted to G^m5^CGC), or M.HpaII (CCGG converted to C^m5^CGG), respectively, by *in vitro* enzymatic modification and the modified plasmid DNAs were subsequently digested by Esp638I. Supplementary Figure S5 shows that unmodified pBR322 and pBRFM were poor substrates for Esp638I digestion (although some nicked circular DNA appeared after digestion). However, Esp638I was able to cleave after *in vitro* modification with M.CviPI or M.SssI. This suggested that a new recognition site was involved. Following run-off sequencing of M.CviPI (Supplementary Figure S5, panel B) and M.SssI (not shown) modified substrates digested with Esp638I, the cut site was shown to be GCN ↓ NGC. To further confirm the recognition sequence and cut sites, phage XP12 DNA was digested by Esp638I and ligated to pUC19. The insert in each plasmid was sequenced and mapped back to the XP12 genome sequence. The cut sites were analyzed using Weblogo (http://weblogo.berkeley.edu/logo.cgi) and gave the consensus sequence as GCS ↓ SGC (relaxed sites GCN ↓ NGC or RCN ↓ NGY with at least four ^m5^C in the two strands) (Fig. S5C). Supplementary Figure S6 shows two examples of Esp638I cut sites (GCG ↓ CGT and GCC ↓ CGC). Since Esp638I prefers to cut GCCCGC sites which potentially overlap with three CpG dinucleotides (cGCCCGCg) and require multiple ^m5^C for efficient cleavage, this enzyme may find a use to study altered methylation patterns in eukaryotic genomes. PROMALS3D multiple sequence alignment of Esp638I homologs (with 50% to 90% aa sequence identity) showed the predicted active site residues E---D-X_(12)-_QxK similar to those of *E. coli* Mrr^29^ (Supplementary Figure S7). Esp638I homologs are present in many sequenced *Pseudomonas* strains including human pathogen *P. aeruginosa*. The sequence specificity and ^m5^C requirement remain to be investigated for these Esp638I homologs. The restriction endonuclease GlaI (R^m5^CGY)^5^ whose gene remains to be identified, may fall into this group of enzymes.

### Star activity or altered specificities of a few BisI homolog enzymes

The BisI homolog enzyme Bth171I partially digested the duplex oligos (G^m5^CWG^m5^C) and fully digested phage XP12 DNA (data not shown). However, pBR322 (containing C^m5^CWGG sites due to M.Dcm modification) was partially digested by Bth171I and Bce1273I as well as Sve396I at high enzyme concentrations (approximately 1–5 μg enzyme to cut 1 μg DNA) (Supplementary Figure S8). To find out what other sites were cleaved under star conditions we performed run-off sequencing of digested pBR322. Figure S8B shows a consensus recognition sequence and cut site (RG ↓ NCY) derived from run-off sequencing for Bce1273I-digested DNA. However, only two sites were completely cut (Supplementary Figure S8C), while the remaining sites were partially digested. Because RGNCY sites are not modified in pBR322, this result suggests that Bce1273I may have lost some of its specificity for ^m5^C modification.

We performed run-off sequencing of Bth171I-digested pBR322. Four cut sites are shown in Supplementary Figure S9A. The consensus recognition sequence and cut sites (RS ↓ NSY) are shown in Figure S9B. The cut sites were similar to those derived from Bce1273I, except that Bth171I generated more complete digestion. It is noted that M.Fnu4HI-modified site G^m5^CNGC is a subset of RSNSY sites and the modified site is cleaved by Bth171I at a low efficiency.

Sve396I prefers to cleave GCNGC sites with three to four ^m5^C. But its star activity at high enzyme concentration can cut unmodified sites at GC ↓ G*A*C (data not shown). To reduce star activity on unmodified sites for the BisI family enzymes, it is best to perform restriction digestion in high salt buffer (NEB buffer 3.1 or buffers with 100 mM KCl or NaCl).

## Discussion

In the work reported here at least three subgroups of BisI family enzymes can be identified from digestion of pBRFM, phage XP12, ^m5^C-modified duplex oligos, T4gt, and pBR322 (M.Dcm^+^) DNA. They display different requirement for the number of modified cytosines in the recognition sequence GCNGC (two to four ^m5^C; three to four ^m5^C, or all four ^m5^C). In some cases, GCWGC modified sites are cleaved more efficiently than GCSGC. Two BisI homologs Bce1273I and Bth171I have degenerate specificities and cut the unmodified sites RG ↓ NCY and RS ↓ NSY in pBR322. A distant BisI homolog, Esp638I displays a unique specificity GCS ↓ SGC (relaxed site RCN ↓ NGY, at least four ^m5^C), generating blunt-ended fragments. Some distantly related BisI family enzymes and Esp638I homologs may have evolved into other specificities.

### Coexistence of BisI endonuclease with C5 MTases

One inactive C5 MTase is adjacent to the BisI restriction gene and a second active C5 MTase located at a distance with the specificity of C^m5^CWGG (the methylome study of the *B*. *subtilis* T30 genome will be reported elsewhere (SYX, Boitano M, Clark TA, Fomenkov A, Guan N, RJR, unpublished result). The active C5 MTase (a M.Dcm-like specificity) is located next to an inactive PLD-family endonuclease. Thus, BisI can coexist with a C5 MTase when the modified site displays a different specificity. Apparently, the presence of a Type I MTase (HsdM/HsdS) in a Type I R-M system that generates ^N6m^A in the *B*. *subtilis* T30 genome would not cause a self-restriction problem. Four other sequenced *Bacillus* genomes contain a gene identical to the BisI restriction gene (REBASE). Similar to the BisI gene organization, the predicted restriction gene is located next to a putative C5 MTase (M.BsuW23 ORF9675P, M.BsuNR231 ORF2772P, M.Bsu231 ORF9920P, M.Bsp663 ORF5699P, identical to the inactive C5 MTase in the *B*. *subtilis* T30 genome). But most of BisI homolog enzymes do not have a companion C5 MTase in close proximity, suggesting that the BisI-like restriction genes could be acquired alone by bacterial horizontal gene transfer mechanisms.

### Targeting a particular GCNGC site

Some BisI homologs can cleave hemi-methylated DNA with two ^m5^C on one strand (i.e. top strand G^m5^CNG^m5^C; bottom strand unmodified). This enzyme property could be utilized to target a particular GCNGC site by hybridization of a single-stranded modified oligo (18–24 nt long), thus creating a strand-specific nick at the targeted site. If two modified oligos are used to target both strands of a particular GCNGC site, then dsDNA cleavage could be achieved (SKD, unpublished results).

### Bce1273I and Bth171I

Although Bce1273I and Bth171I cleaved frequent sites, they were expressed in the absence of a protective methylase. It is possible that low specific activity or fusion to intein and CBD domains reduced the toxic effect on expression in *E. coli*. It is known that low activity mutants of BamHI, BsoBI, and EcoRI are tolerated in *E. coli*. Although a lot of protein is required to cut pBR322 into small fragments, Bth171I appears to be the most frequent cutter that might be useful to digest genomic DNA into small pieces for library construction. In the shotgun sequence of *B. cereus* AH1273 genome fragment, the Bce1273I gene is located next to a putative phage major capsid protein and a Tyr recombinase/integrase and thus Bce1273I is probably associated with a mobile genetic element. In the whole genome sequence of *B. thuringiensis* BMB171, the Bth171I restriction gene is also associated with a prophage due to its close proximity to a Tyr recombinase (integrase), phage terminase small and large subunits, and capsid morphogenesis proteins. It is speculated that Bth171I may represent an evolutionary intermediate of Type II and Type IIM REase. Further mutation(s) and ^m5^C-modified phage infection/selection may yield more active modification-dependent variants. Alternatively, it may become a more active Type II REase through mutation and natural selection if a companion MTase gene could be acquired through horizontal gene transfer (i.e. the newly acquired MTase modifies the GCNGC site and blocks digestion). The third high possibility is for it to become completely inactive if it does not provide any evolutionary advantage to the host, which might be the case for Bth171I at the current evolutionary stage.

### Other REases recognizing GCNGC, GCWGC, or GCSGC

Another enzyme, only remotely related to BisI, is the recently described EcoBLI that cleaves GCNGC sites containing two to four ^m5^C^30^. More than 30 homologs are present in GenBank with >30% aa sequence identity to EcoBLI by BlastP search.

There are a number of Type IIP REases cleaving unmodified GCNGC sites (Fsp4HI)^31^, GCWGC (ApeKI and TseI), or GCSGC (TauI) sites^7^. The aa sequences of these enzymes and other homologs have very low sequence similarity to the BisI family enzymes (less than 15% identity), suggesting that Type IIP enzymes cleaving BisI-related sites evolved independently.

## Methods

Synthetic gene blocks (gblock) with optimized *E. coli* codons were synthesized by IDT (Coralville, Iowa) and cloned into the NdeI and XhoI sites of the pTYB1 (NEB) expression vector by using a Gibson assembly kit (NEB). The gblock coding for Rfl17I (with an N-terminal 6xHis tag) was cloned into the expression vector pBAD241 (flanked by NdeI and HindIII, the target gene under the control of P_BAD_ promoter, inducible by arabinose) (N.Guan, unpublished). After isolation of plasmids containing the correct size inserts, the inserts were sequenced to confirm the correct sequences were present coding for the wild-type REase. IPTG-induction (0.5 mM IPTG final concentration) of late-log ER2566 cells (OD_590_ = 0.5 to 0.6) harboring appropriate plasmids was carried out at 16 °C to 18 °C overnight for protein production. The same procedure was followed for the purification of intein-CBD-BisI homolog fusions from chitin columns and DTT cleavage was used to release the target proteins^23^. After protein elution from chitin columns, eluents were concentrated using Amicon Ultra-15 centrifugal filter units and protein was diluted into a storage buffer (0.2 M NaCl, 20 mM Tris-HCl, pH 7.8, 1 mM DTT, 50% glycerol). The partially purified REases were further diluted to 1 mg/ml with NEB restriction enzyme diluent buffer A (50 mM KCl, 10 mM Tris-HCl, pH 7.4, 0.1 mM EDTA, 1 mM DTT, 200 μg/ml BSA, 50% glycerol) and stored at −20 °C. His-tagged Rfl17I enzyme was partially purified from Ni-NTA affinity columns (Superflow Ni-agarose beads, Qiagen). Protein was concentrated and storage buffer was exchanged as described above. After chitin column purification, BisI and Vsp586I endonucleases were further purified by chromatography using a Heparin column (HiTrap-Heparin, 5 ml, GE Life Sciences). BsiI and Vsp586I were eluted by a salt gradient (50 mM to 1 M NaCl, 20 mM Tris-HCl, pH 7.5, 1 mM DTT, 1 mM EDTA). Peak fractions with BisI and Vsp586I were diluted into the storage buffer described above. Regardless of the source of the original bacterial strains, the BisI family enzymes were assayed at 37 °C for restriction activity. Restriction fragments were purified by spin columns (Qiagen) and subjected to BigDye terminator cycle sequencing (ABI) with specific primers to determine the recognition sequence and cut site.

Phage XP12 genomic DNA (^m5^C) was a gift from P. Weigele (NEB) and T4gt gDNA (^hm5^C) was provided by Y. Zheng (NEB). Modified plasmid pUC19-Fnu4HIM (modified sites G^m5^CNGC, abbreviated as pUCM) was a gift from R. Morgan (NEB). To increase *fnu4HIM* gene expression, a strong ribosome binding site (GGAGGTtaataa) was engineered in front of the gene and cloned into pBR322 (BamHI-SphI) under constitutive expression from the Tc promoter (this plasmid pBR322-Fnu4HIM abbreviated as pBRFM is completely resistant to Fnu4HI digestion). PBRFM contains two types of modified sites: G^m5^CNGC (two ^m5^C, the cytosine opposite of the underlined G is also modified) and G^m5^CNG^m5^CNGC (three ^m5^C), which can serve as a substrate for BisI family enzymes cleaving GCNGC with two or three ^m5^C. It is a poor substrate for BisI homologs requiring four ^m5^C for efficient cleavage.

5′ FAM labeled ^m5^C modified oligos were synthesized by IDT. After restriction digestion the cleavage products were analyzed by PAGE (15–20% PAG or PAG-urea gels). SYBR Gold stained or non-stained gels were analyzed on a Typhoon fluorescence imager (GE Life Sciences). The following duplex oligos were used for restriction activity assays:

Top strand 5′ (FAM)-AGATCCAAGCTTGAATTC **G** ^**m5**^**CAG** ^**m5**^**C** CATATGGCTCT 3′ (two ^m5^C in this oligo, BisI recognition sequence shown in bold).

#1. Bottom strand 5′ AGAGCCATATG **G** ^**m5**^**CTG** ^**m5**^**C** GAATTCAAGCTTGGATCT 3′ (two ^m5^C).

#2. Bottom strand 5′ AGAGCCATATG **G** ^**m5**^**C TGC** GAATTCAAGCTTGGATCT 3′ (one ^m5^C-internal modified cytosine).

#3. Bottom strand 5′ AGAGCCATATGGCTGCGAATTCAAGCTTGGATCT 3′ (no ^m5^C).

Duplex oligos were formed by mixing two ssDNAs as following (heating at 95^o^C for 5 min and cooled down at room temperature):

Top strand + #1 oligo = four ^m5^C, G ^m5^C AG ^m5^C + G ^m5^C TG ^m5^C

Top strand + #2 oligo = three ^m5^C, G ^m5^C AG ^m5^C + G ^m5^C TGC

Top strand + #3 oligo = two ^m5^C, G ^m5^C AG ^m5^C + GCTGC (hemi-methylated, top-strand only). In some experiments, ssDNA was degraded by the addition of 10 U of *E. coli* exonuclease I (10 min at 37^o^C) after restriction digestion of the duplex oligos.

A PCR fragment containing the ORF Bis30_20225 (a putative HNH endonuclease) was cloned in pTYB1 (NdeI-XhoI) and expressed in *E. coli*^23^. Its gene product (gp) was purified from a chitin column. Plasmids pBR322 and pBRFM were used as substrates for the nicking activity assay. Cleavage (nicking) sites were determined by DNA run-off sequencing.

NhoI unit definition: 1 unit of the enzyme is the amount of protein to digest 1 μg of phage XP12 DNA into products of less than 200 bp in NEB buffer 2.1 in 1 h.

BisI and Bce95I unit definition: 1 unit of the enzyme is the amount of protein required to digest 1 μg pBRFM DNA to completion in NEB buffer 2.1 in 1 h.

## Additional Information

**How to cite this article**: Xu, S.-Y *et al*. Expression and purification of the modification-dependent restriction enzyme BisI and its homologous enzymes. *Sci. Rep*. **6**, 28579; doi: 10.1038/srep28579 (2016).

## Supplementary Material

Supplementary Information

## Figures and Tables

**Table 1 t1:** Active BisI homolog enzymes that cut GCNGC sites with two to four ^m5^C.

REase^a^	GenBank accession #	Bacterial strain	# of aa & aa seq identity to BisI^b^	Activity on pBRFM (2–3 ^m5^C)	Cut sites & Comment^c^
BisI	AJW87312	*B. subtilis* T30	168 aa (100%)	Yes	Prototype, 2–4 ^m5^C in GCNGC
Rfl17I	WP_019680669	*Ruminococcus flavefaciens*	167 aa (57.1%)	Yes	2–4 ^m5^C
BceYI	EJR31148	*Bacillus cereus* VD078	164 aa (53.5%)	Yes	2–4 ^m5^C
Bce95I	EEL18168	*Bacillus cereus* 96/8201	170 aa (42.1%)	Yes	2–4 ^m5^C
Vsp586I	EEY99140	*Vibrio* sp. RC586	152 aa (41.7%)	Yes	2–4 ^m5^C
KasKI (Pk2)	WP_035896512	*Kluyvera ascorbata*	158 aa (27.8%)	Yes	2–4 ^m5^C
CbuDI (PK1)	WP_003424307	*Clostridium butyricum* DKU-01	162 aa (24.3%)	Yes	2–4 ^m5^C (star activity on Dcm+ λ)^d^
Pps170I	GAC38390	*Paraglaciecola psychrophila* 170	157 aa (23.6%)	Yes	2–4 ^m5^C
VspHI	EKM27722	*Vibrio cholera* HENC-03	150 aa (21.4%)	Yes	2–4 ^m5^C (low activity on ^m5^C oligos)
MspAK21I (PK11)	WP_036129618	*Marinobacter* sp. AK21	152 aa (21.4%)	Yes	2–4 ^m5^C (star activity on Dcm+ λ)
AspTB23I (PK12)	WP_019481883	*Arthrobacter sp*. TB 23	162 aa (16.5%)	Yes	2–4 ^m5^C (star activity on Dcm+ λ)
LsaM18I (PK13)	WP_017793998	*Leucobacter salsicius*	172 aa (16.5%)	Yes	2–4 ^m5^C (star activity on Dcm+ λ)

^a^All enzymes listed here are active in digestion of phage XP12 DNA (all ^m5^C).

^b^Pairwise sequence alignment by EMBOSS Needle server (gap penalty = 10.0, extend penalty = 0.5). List of enzymes in descending order of amino acid sequence identity to BisI.

^c^All enzyme cut sites were determined by run-off sequencing of the enzyme-digested pBRFM substrate. d. star activity was observed in NEB buffer 2.1.

**Table 2 t2:** A list of active BisI homolog enzymes that prefer to cut GCNGC sites with three to four ^m5^C.

REase	GenBank accession # or locus tag	Bacterial strain	# of aa & aa seq identity to BisI^b^	Activity on pBRFM (2–3 ^m5^C)	Cut sites & Comment^c^
Eco15I	EIL45146	*E. coli* 541-15	290 aa (26.2%, N-terminal 160 aa)	Yes	3–4 ^m5^C > 2 ^m5^C
Sde240I (PK7)	WP_041325806	*Saccharophagus degradans*	162 aa (24.3%)	Yes	3–4 ^m5^C > 2 ^m5^C
Pan13I	WP_014606222	*Pantoea ananatis* PA13	157 aa (24.0%)	Yes	3–4 ^m5^C > 2 ^m5^C
Pru4541I	EFB72318	*Providencia rustigianii* DSM 4541	173 aa (23.9%)	Yes (3 ^m5^C sites)	3–4 ^m5^C > 2 ^m5^C
AlaI (PK4)	WP_026374325	*Agrococcus lahaulensis*	160 aa (23.0%)	Yes	3–4 ^m5^C > 2 ^m5^C
Sve396I	EFQ58914	*Streptococcus vestibularis* F0396	164 aa (22.3%)	Yes (3 ^m5^C sites)	3–4 ^m5^C 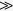 2 ^m5^C
SqiI^a^ (Pk9)	WP_026758869	*Sediminimonas qiaohouensis*	161 aa (22.3%)	No	4 ^m5^C 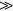 3 ^m5^C
Dsp20IU (PK5)	KDB03925	*Defluviimonas* sp. 20V17	161 aa (22.1%)	Yes	3–4 ^m5^C > 2 ^m5^C. prefer GCWGC
NhoI	CCF83679	*Nitrolancetus hollandicus* Lb	169 aa (21.9%)	Yes (3 ^m5^C sites)	3–4 ^m5^C 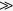 2 ^m5^C
SmaAUI	EMI48892	*Stenotrophomonas maltophilia* AU12-09	168 aa (20.4%)	Yes	3–4 ^m5^C > 2 ^m5^C
MbaR4I (PK3)	WP_017202926	*Microbacterium barkeri*	190 aa (17.2%)	Yes	3–4 ^m5^C > 2 ^m5^C

^a^SqiI requires four ^m5^C in GCWGC (4 ^m5^C 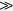 3 ^m5^C) for activity. The cut site of SqiI and MbaR4I was inferred from digestion of modified duplex oligos.

^b^Pairwise sequence alignment by EMBOSS Needle server (gap penalty = 10.0, extend penalty = 0.5). List of enzymes in descending order of aa sequence identity to BisI.

^c^Cut sites were determined by run-off sequencing of enzyme-digested pBRFM substrate. The cleavage efficiency of the modified site (3–4 ^m5^C) was carried out on duplex oligos. The cut sites of Eco15I, Pan13I, Pru4541I, SmaAUI, Sve396I, and NhoI were determined by run-off sequencing of enzyme-digested pBRFM substrate (GCNGC sites with two or three ^m5^C).

**Table 3 t3:** Unique specificity and enzymes with degenerate specificities on unmodified DNA.

REase	GenBank accession #	Bacterial strain	# of aa & aa seq identity to BisI^a^	Activity on pBRFM (2–3 ^m5^C)	Cut sites & Comment^b^
Bce1273I	EEL90735	*Bacillus cereus* AH1273	164 aa (45.8%)	No	Activity on pBR322 RGNCY
Bth171I	WP_001138855	*Bacillus thuringiensis* BMB171	164 aa (42.4%)	Yes	Activity on pBR322 RSNSY
Esp638I (PK14)	ABP61776	*Enterobacter sp*. 638	166 aa (23.2%)	No	Unique specificity GCNNGC or RCNNGY (4–6 ^m5^C)

^a^Pairwise sequence alignment by EMBOSS Needle server (gap penalty = 10.0, extend penalty = 0.5).

^b^The cut sites of Bce1273I and Bth171I were determined by run-off sequencing of enzyme-digested pBR322. BceLI (WP_000093752) is similar to Bce1273I and Bth171I with degenerate (relaxed) specificity. Esp638I cut site was determined by run-off sequencing of M.CviPI- or M.SssI-modified pBR322 DNA. Esp638I cut site was also confirmed by cloning and sequencing of Esp638I-digested phage XP12 DNA.
