# Supplementary material for: Expression and purification of the modification-dependent restriction enzyme BisI and its homologous enzymes
Source: Sci Rep. 2016 Jun 29;6:28579. doi: 10.1038/srep28579 (PMC4926085; doi:10.1038/srep28579)

Supplementary Information

(Supplementary Table S1, Supplementary Figures S1 to S9)

Expression and purification of the modification-dependent restriction enzyme BisI and its homologous enzymes

Shuang-yong Xu[1*], Pernelle Klein[1#], Sergey Kh. Degtyarev[2], Richard Roberts[1*]

1. New England Biolabs, Inc. 240 County Road, Ipswich, MA 01938, USA
2. SibEnzyme Ltd. 2/12 Ak. Timakov Street, Novosibirsk 630117, Russia

*Corresponding authors:

Email: xus@neb.com

Telephone: 1-978-380-7287

Fax: 1-978-921-1350

Or

Email: roberts@neb.com

Telephone: 1-978-380-7405

Fax: 1-978-921-1350

# Current address:

École supérieure de biothechnologie Strasbourg (ESBS), Université de Strasbourg
300 boulevard Sébastien Brant, CS 10413, F-67412 Illkirch. France.

Supplementary Table S1. A list of low activity or inactive BisI homologs

**Clone isolate**	**GenBank accession #**	**Bacterial strain (genome)**	**Protein size, aa seq identity to BisI**	**Activity on plasmid Fnu4HIM^{+} (2-3 ^{m5}C)**	**Activity on phage XP12 (^{m5}C)**	**Additional comment**
VH4-BceV	EOO35066	*Bacillus cereus* VDM019	212 aa (44%) (M_{58} start codon)	Low activity	??	Poor protein yield (N-terminal 6xHis tag)
XU-Sme	AGA08338	*Sinorhizobium melliloti* GR4	169 (36.1%)	No	No	Low protein yield
XU-Psp	EJM90208	*Pseudomonas* sp. GM67	192 (31.2%)	Low activity	??	Protein made
XU-Bvi	ABO60211	*Burkholderia vietnamiensis* G4	174 (22.5%)	No	No	Low protein yield
XU-Hde	EHB76573 (ATG start codon)	*Hyphomicrobium denitrificans* 1NES1	183 (22.4%)	Low activity	??	Protein made
PK6	EHB76573 (TTG start codon)	*Hyphomicrobium denitrificans* 1NES1	172 (20.7%)	No	No	Protein made
XU-Tsu	AEB13592	*Treponema succinifaciens* DSM 2489	236 aa (20.5%,N-terminus)	No	No	Protein made
PK10	WP_029989486	*Microbacterium paraoxydans*	196 aa (18.6%)	No	No	Poor protein yield

Proteins listed in descending order based on amino acid sequence identity to BisI.
??, not tested for restriction activity on phage XP12 DNA substrate.

Supplementary Figure S1.

SDS-PAGE analysis of partially purified BisI homologs and restriction activity assays on plasmid and phage XP12 DNA substrates.

A. SDS-PAGE analysis of partially purified BisI homolog enzymes (see Tables 1 to 3 for the list). Lanes 1-13, PK #1 to #13 clones (1-CbuDI, 2-KasKI, 3-MbaR4I, 4-AlaI, 5-Dsp20I, 6-PK6, 7-Sde240I, 8-SqiI, 9-PK10, 10-MspAK21I, 11-AspTB23I, 12-LsaM18I, 13-Esp638I). The stars denote the over-expressed and partially purified target proteins.

B. and C. Restriction activity assays on pUC19-Fnu4HIM and phage XP12 DNA, respectively. Lanes 1-13, input enzymes are numbered as in A. Note: in lanes 3 (MbaR4I), 8 (SqiI), and 13 (Esp638I), strong endonuclease activity was detected on phage XP12 DNA only; poor activity (linear or partial nicking) was detected on the modified plasmid substrate. “--”, uncut DNA.

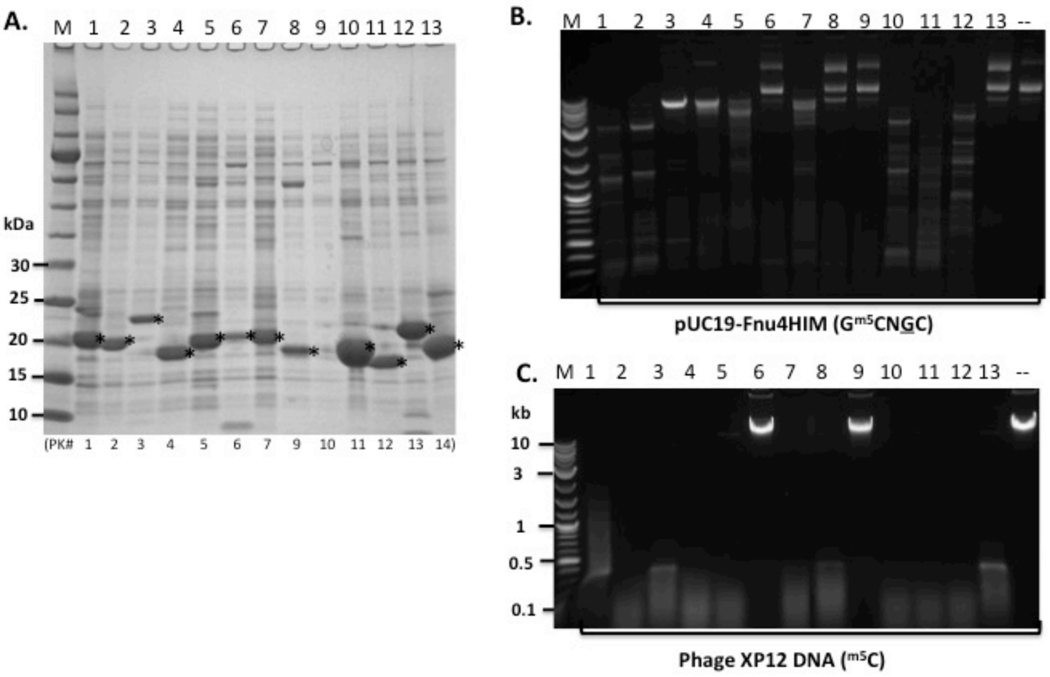

Supplementary Figure S2

Digestion of modified duplex oligos (containing two to four ^{m5}C) by BisI homologs MbaR4I (PK#3), SqiI (PK#9), Esp638I (PK#14), and other enzymes.

A. MbaR4I, SqiI, and Esp638I digested duplex oligos with four, three, or two modified bases ^{m5}C. The substrate (Sub) and product (P1, denoted by a "*") were detected by FAM fluorescence imaging.

B. MbaR4I, SqiI, and Esp638I digested duplex oligos (left panel, four ^{m5}C; right panel, three ^{m5}C) analyzed on a 20% PAG gel. DNA bands were stained by SYBR Gold and detected by fluorescence imaging. "*" indicates the cleavage products P1 (20 bp) and P2 (14 bp). 5 bp, DNA size markers.

C. Hemi-methylated duplex oligos (two ^{m5}C on top strand only, unmodified bottom strand) digested by BisI homolog enzymes. "*", cleavage product P1 as detected by FAM fluorescence imaging. After restriction digestion, 10 U of *E. coli* Exonuclease I was added to degrade any ssDNA.

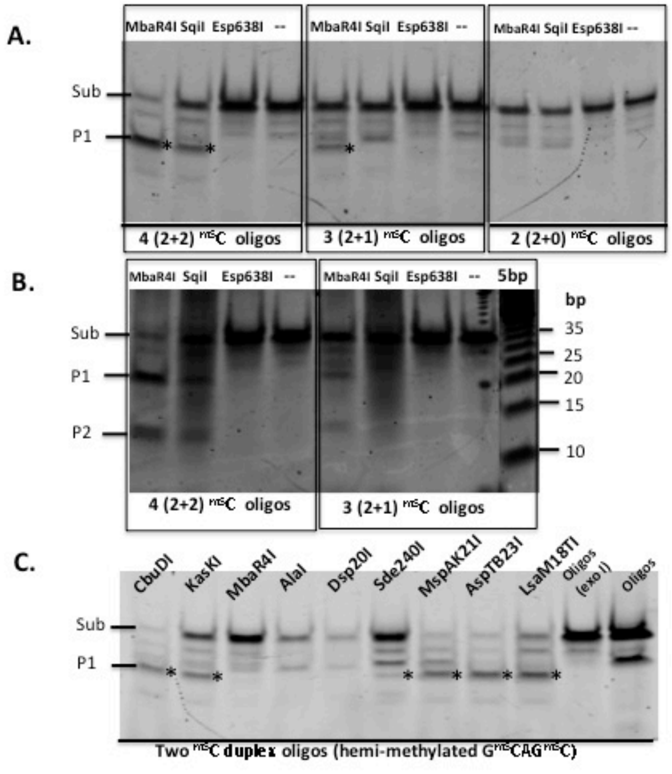

Supplementary Figure S3.

PROMALS3D (http://prodata.swmed.edu/promals3d) multiple sequence alignment for BisI, Bce95I, BceYI-like enzymes (cleavage of two to four ^{m5}C in GCNGC). The sequences in the box are predicted to form a typical REase fold (αβββαβ, the second β strand may be replaced by an α-helix) that contain 3-4 catalytic residues D, E, and K for metal ion (2-3 Mg^{2+}) binding and catalysis. The conserved aa sequence **E**(or D)LG**E**---**ID**AI---RYSI**K**XT---TG(or S)VF-GL(V or M)---Q-F**E(or D)**Y-IIV or variants of this sequence can be found in BisI/Bce95I/BstYI-like enzymes and over 30 hypothetical proteins in sequenced bacterial genomes. Numbers (6 to 9) on top of the aa sequences show the propensity of conservation. "*", predicted catalytic residue candidates.

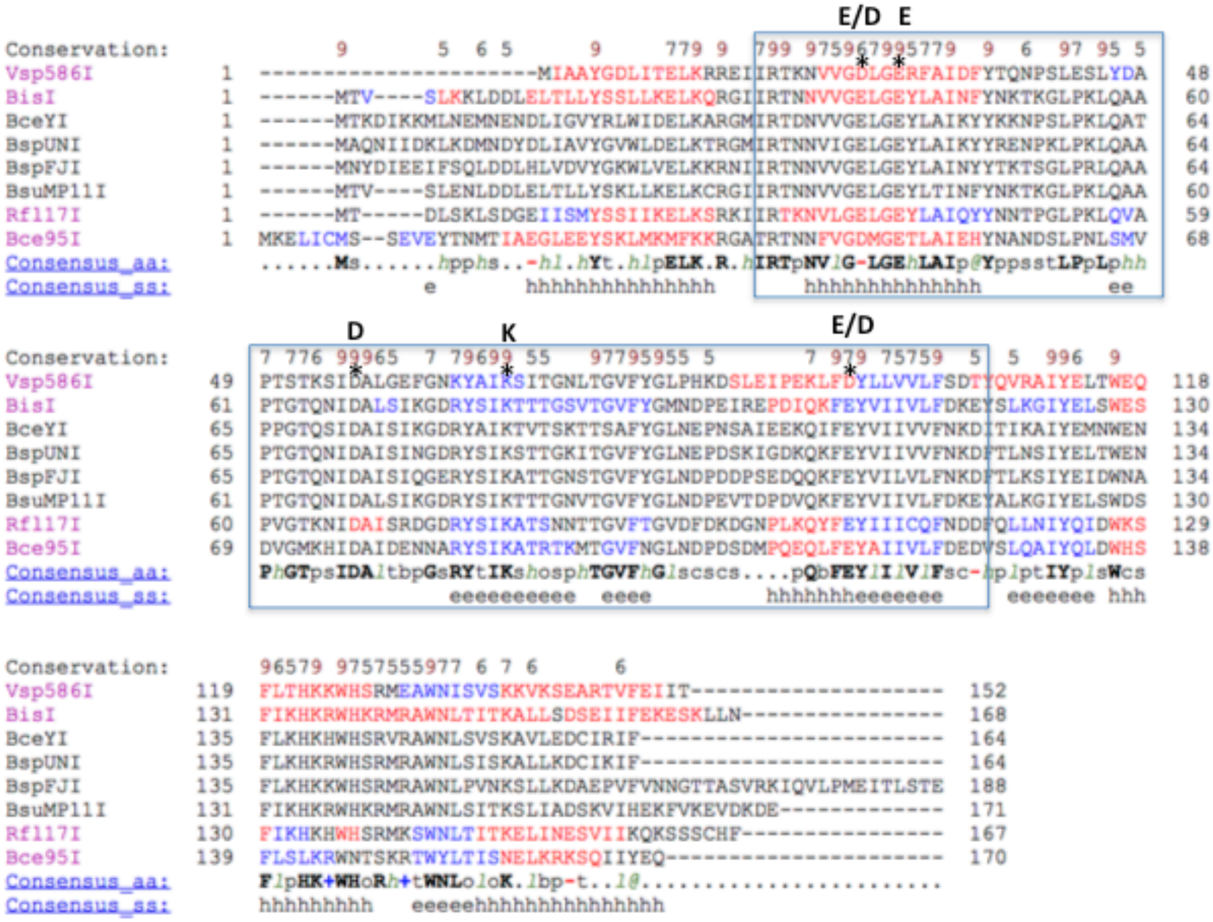

Supplementary Figure S4.

PROMALS3D multiple sequence alignment for NhoI, MbaR4I, SqiI-like enzymes (required three to four ^{m5}C in GCNGC for efficient cleavage). The aa sequences in the box are predicted to form the typical REase fold (αβββαβ) that contains the putative catalytic residues D or E, D-X_{10}-QxK for metal ion (2-3 Mg^{2+}) binding and catalysis (a variant of PD-ExK, Mrr-like catalytic site). "*", predicted catalytic residue candidates.

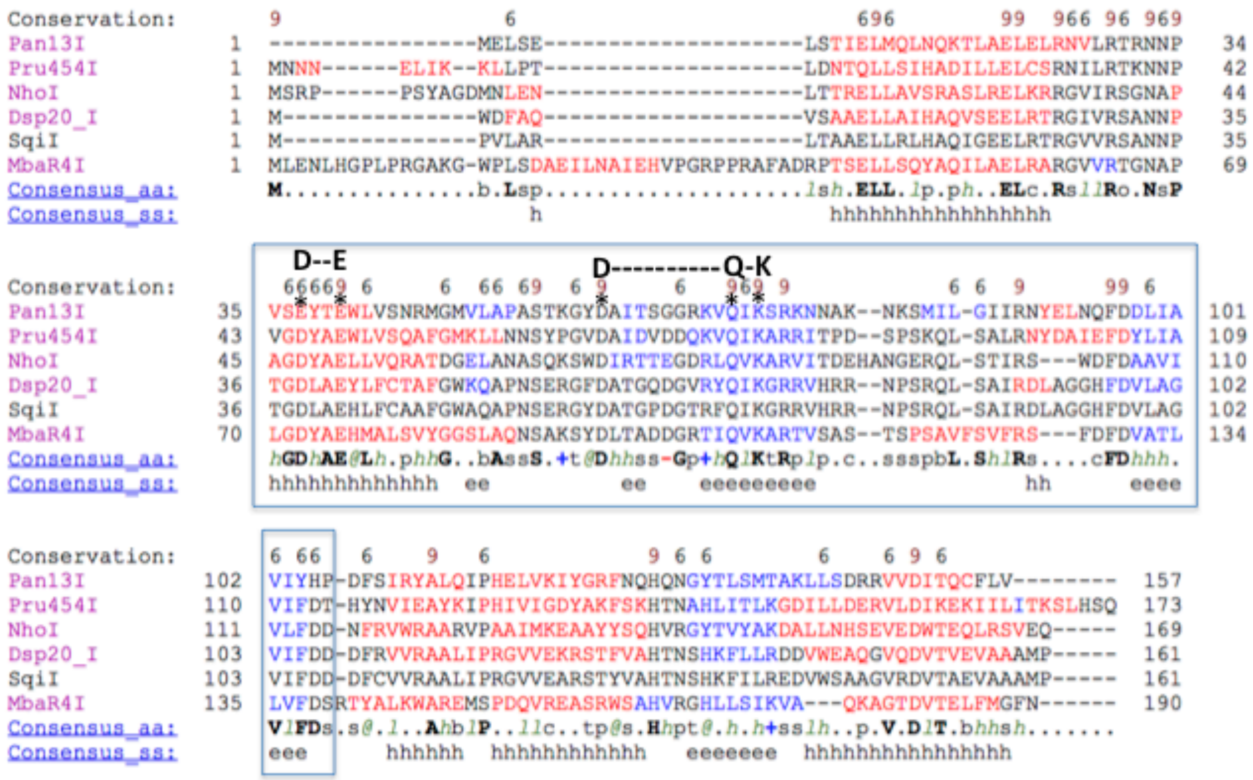

Supplementary Figure S5.

Esp638I digestion of M.CviPI-modified pBR322 and phage XP12 DNAs and a summary of Esp638I recognition sequences and cut sites.

A. Esp638I digestion of pBRFM, pBRFM+ (additionally modified by M.CviPI), M.CviPI-modified pBR322, pBR322, and phage XP12 DNAs.

B. A list of Esp638I recognition sequences and cut sites determined by run-off sequencing of Esp638I-digested, M.CviPI-premodified pBR322 DNA.

C. Consensus recognition sequence (GCN↓NGC or RCN↓NGY) derived from cloned phage XP12 DNA fragments as compiled by Weblogo.

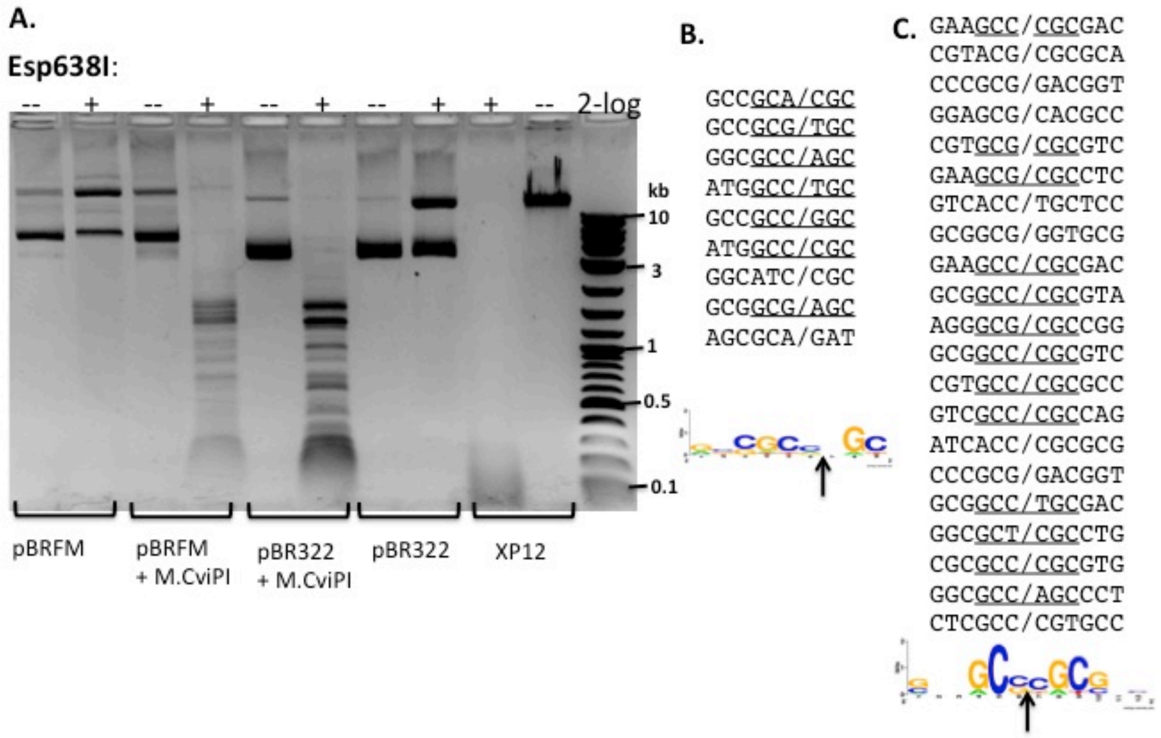

Supplementary Figure S6.

DNA run-off sequencing of Esp638I-digested pBR322 DNA, M.SssI-modified (left sequence panel), M.CviPI-modified (right sequence panel). Both the forward and reverse strands were shown as compared to undigested templates. GCAGC (indicated by a green bar) was not cut by Esp638I.

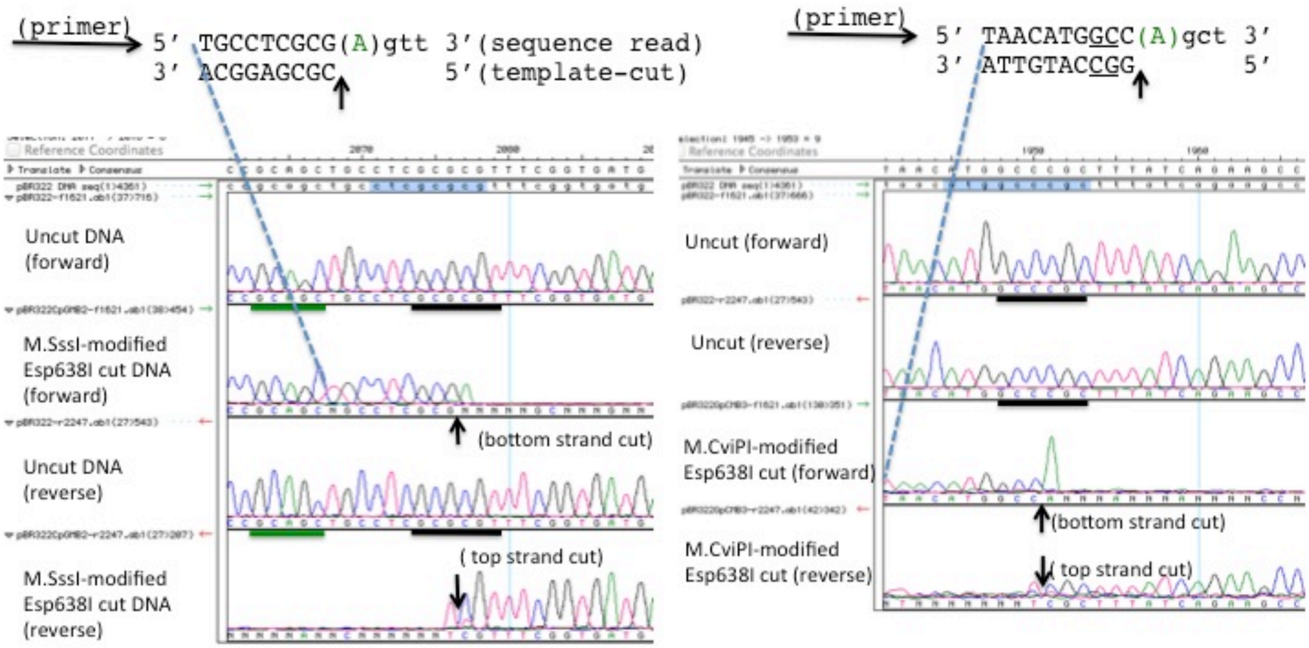

Supplement Figure S7.

PROMALS3D multiple sequence alignment for Esp638I-related enzymes (with 50% to 90% aa sequence identity to Esp638I). The function and specificity of these homologous enzymes remain to be analyzed. The sequences in the box are predicted to form the typical REase fold (αβββαβ) that contains the catalytic residues E-$X_{19\text{-}22}$-D-X_{12}-QxK for metal ion (2-3 Mg^{2+}) binding (a variant of the PD-D/ExK catalytic site). The bacterial sources and GenBank accession numbers are: Esp638I (WP_015960106), WP_058676714 (*Enterobacter hormaechei*), WP_049016189 (*Citrobacter koseri*), WP_001592532 (*Enterobacteriaceae* sp.), WP_032723186 (*Enterobacter aerogenes*)), WP_040219152 (*Klebsiella pneumonia*), WP_058702817 (*Pantoea stewartii*), EGH79119 (*Pseudomonas syringae* pv. aptata str. DSM 50252), WP_043042189 (*Pseudomonas fluorescens*), WP_054068722 (*Pseudomonas amygdali*), WP_049885553 (*Pseudomonas aeruginosa*), WP_039487727 (*Pectobacterium carotovorum*). "*", predicted catalytic residue candidates (E---D---Q-K).

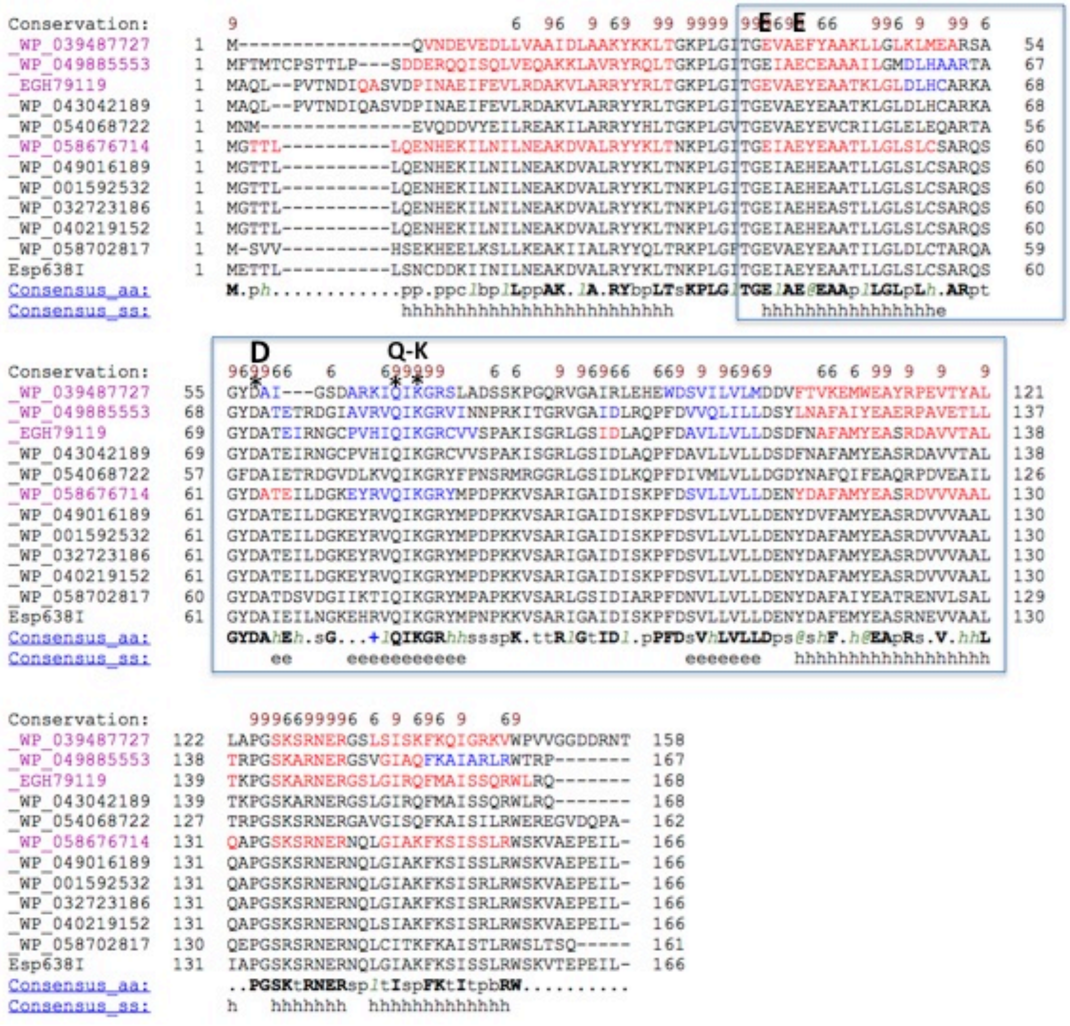

Supplementary Figure S8.

Star activity or altered specificities of three BisI family enzymes Bth171I, Bce1273I, and Sve396I in digestion of pBR322 (M.Dcm^{+}) and DNA run-off sequencing of Bce1273I-digested pBR322.

A. Agarose gel analysis of cleavage products (1 μg of pBR322) digested by 1 or 5 μg of REase as indicated above each lane. "--", uncut DNA, 2-log, 2-log DNA ladder.

B. Bce1273I cut sites derived from run-off sequencing and the consensus recognition sequence (RG↓NCY, relaxed sites RS↓NSY) compiled by Weblogo.

C. Two examples of run-off sequence of Bce1273I cut sites (AG↓TCC and AG↓TCT) were shown.

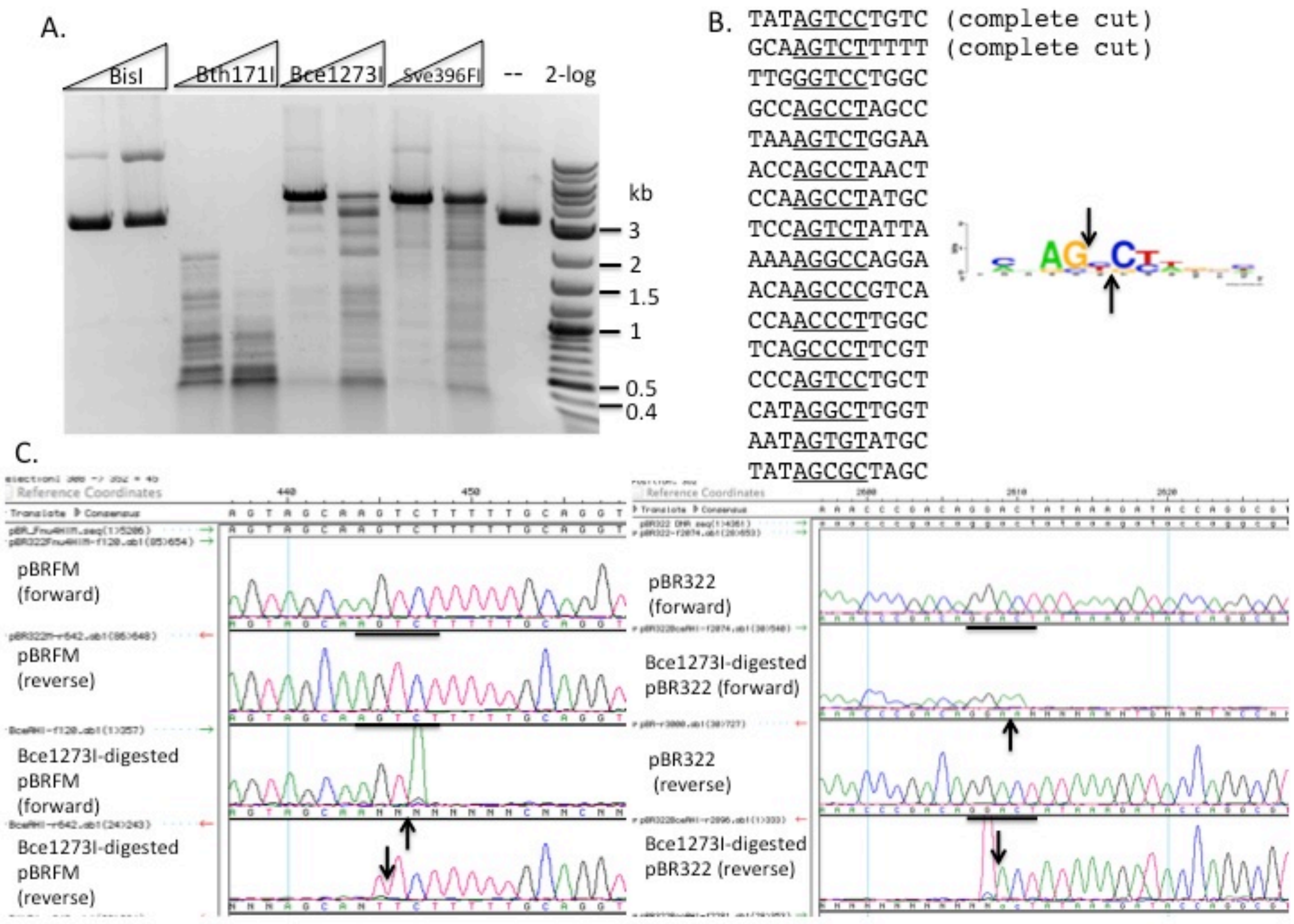

Supplementary Figure S9.

Run-off sequencing of Bth171I-digested pBR322 (M.Dcm+) plasmid and the consensus sequence compiled by Weblogo.

A. Four examples of run-off sequence of Bth171I cut sites (GG↓CCT, GC↓CCT, AG↓CCT, and AC↓CCT).

B. Bth171I cut sites compiled by Weblogo to generate a consensus sequence RS↓NSY. The top five sites were digested completely (complete run-off) and the rest of the sites were partially digested to give partial run-off traces.

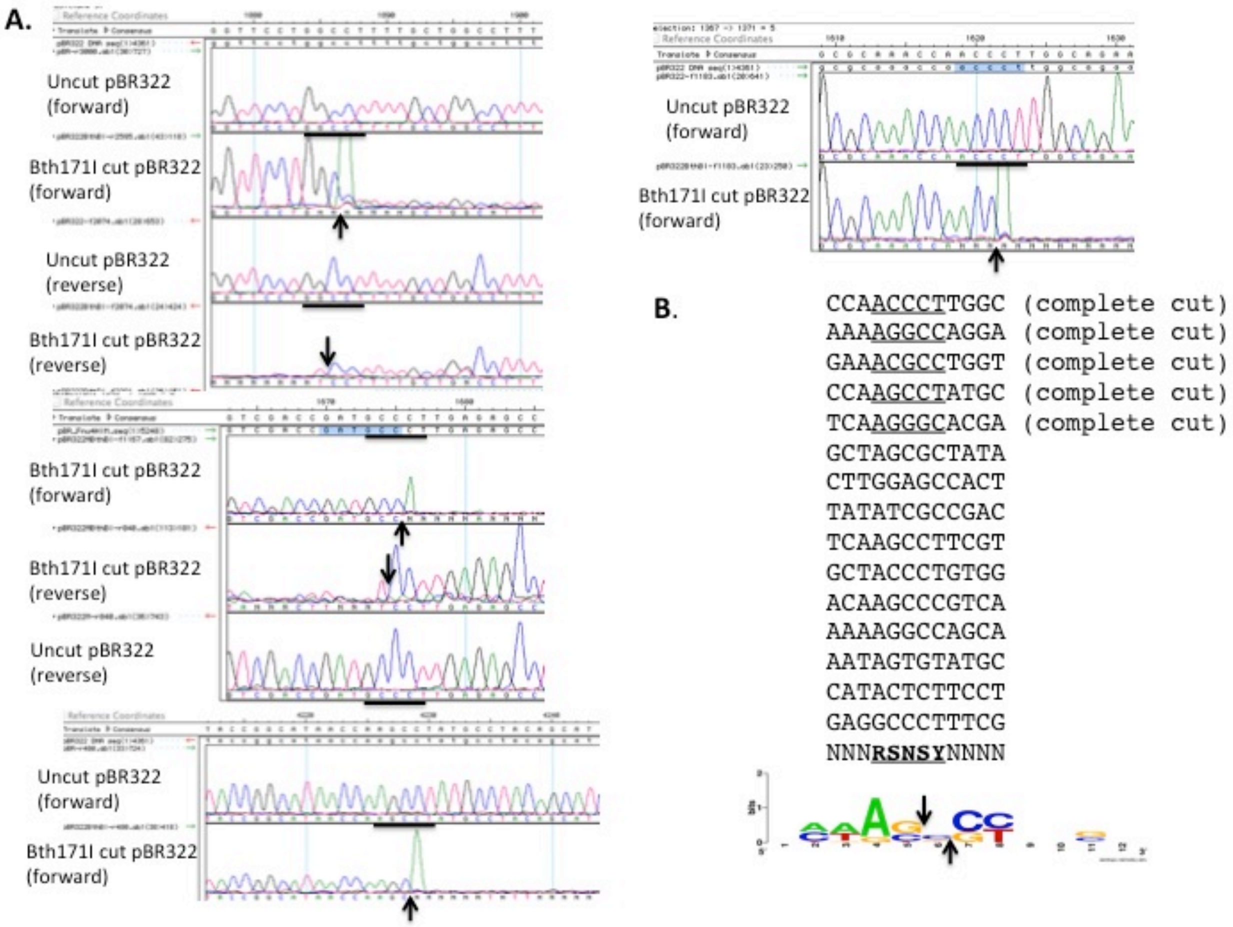

Supplement: Supplementary Information [file srep28579-s1.pdf]
